# Shenmai injection for the treatment of cancer-related fatigue in advanced non-small cell lung cancer patients undergoing chemotherapy: study protocol for a randomized controlled trial

**DOI:** 10.1186/s13063-018-2845-7

**Published:** 2018-09-04

**Authors:** Yushu Zhou, Baiming Zhao, Wanyin Wu, Xiaobing Yang, Shunqin Long, Hong Deng, Wenfeng He, Guiya Liao, Qiuping Li, Zhen Xie

**Affiliations:** 1 Department of Oncology, Guangdong Provincial Hospital of Traditional Chinese Medicine, No. 111 Dade Road, Guangzhou, 510120 Guangdong China; 2 Department of TCM, The Third Affiliated Hospital of Sun Yat-Sen University, No. 600 Tianhe Road, Guangzhou, 510630 Guangdong China

**Keywords:** Traditional Chinese medicine, Cancer-related fatigue, Non-small cell lung cancer, Shenmai injection, Randomized controlled trial

## Abstract

**Background:**

Cancer-related fatigue (CRF) is the most common symptom in patients with advanced non-small cell lung cancer (NSCLC) undergoing treatment with chemotherapy. However, evidence upon which to base management strategies is scarce. Traditional Chinese Medicine (TCM) has been shown to be beneficial to patients with CRF. Chinese herbal injections should be administered under an evidence-based approach. This trial aims to assess the efficacy and safety of the addition of the Shenmai injection (SMI) to conventional therapy for CRF in NSCLC patients undergoing chemotherapy.

**Methods/design:**

The study is a two-group, prospective, randomized controlled trial (RCT) designed to evaluate the efficacy and safety of SMI for CRF NSCLC patients undergoing chemotherapy. Eligible participants will be randomized to either a treatment group receiving a 5-day Shenmai injection regimen plus conventional therapy or a control group receiving only conventional therapy. The primary outcome is fatigue, assessed using severity scores from the Functional Assessment for Chronic Illness Therapy-Fatigue (FACIT-F) measurement system. Secondary outcomes include symptom distress scores, depression, sleep disorders, quality of life, and levels of immunologic indicators. Assessments will be carried out at baseline and on day 5 (the end of the intervention).

**Discussion:**

This study can provide evidence to support clinical decision-making in the management of CRF in NSCLC patients undergoing chemotherapy in a way that can be scaled up and used throughout China.

**Trial registration:**

Chinese Clinical Trial Registry (chictr.org.cn), ChiCTR-INR-17013737. Registered on 6 December 2017.

**Electronic supplementary material:**

The online version of this article (10.1186/s13063-018-2845-7) contains supplementary material, which is available to authorized users.

## Background

Lung cancer is the leading cause of cancer-related mortality worldwide [1]. Chemotherapy remains the primary treatment for non-small cell lung cancer (NSCLC) patients. A history of chemotherapy was independently associated with severe cancer-related fatigue (CRF) in patients with various types of advanced cancer [2]. Chemotherapy has considerable toxicity, which is a significant factor in the severe fatigue often experienced by cancer patients. Fatigue, in turn, can become a dose-limiting factor during chemotherapy.

CRF has been defined by a panel of the National Comprehensive Cancer Network (NCCN) as a persistent, subjective sense of tiredness related to cancer or cancer treatment that interferes with usual functioning [3]. One of the characteristics of CRF that distinguishes it from other types of fatigue is that it is not relieved by sleep or rest [3–5], and patients report being “unusually” or overwhelmingly tired [3–6].

Cancer patients undergoing aggressive therapy and those with advanced disease often experience clusters of severe symptoms led by fatigue. CRF most commonly occurs with other symptoms, such as depression, anxiety, pain, sleep disorder, and loss of functional status [7]. Overall 50–90% of people with cancer experience fatigue [8–10]. CRF profoundly impacts on a person’s physical, emotional, and mental well-being [11]. It has a clear negative impact on the quality of life of people with cancer and their ability to maintain their usual personal, professional, and social relationships [12].

The development of fatigue occurs alongside other symptoms, such as pain, distress, poor appetite, drowsiness, and disturbed sleep. It provides a rationale for studying the role of inflammation as a common mechanism underlying the production of multiple symptoms, including fatigue.

The hypothesis that activation of the proinflammatory cytokine network induces fatigue has been under investigation since the 1990s. Dysregulated inflammation and its toxic downstream effects are believed to constitute a significant biological basis for CRF and other symptoms [13–15]. Preclinical research on immune-to-brain communication pathways in the peripheral immune system indicates that proinflammatory cytokines, primarily interleukin (IL)-1β and tumor necrosis factor (TNF)-α, send signals to the brain that promote symptoms associated with sickness, including fatigue, disturbed sleep, and depressive symptoms in vulnerable individuals. TNF-α has been found to be a prognostic inflammatory biomarker of persistent fatigue [16]. TNF-α signaling has been associated with postchemotherapy fatigue in patients with early-stage breast cancer [17].

Despite the frequency of CRF in cancer patients and various pharmacologic and nonpharmacological approaches that have been studied, it still remains underdiagnosed and undertreated, partly because of limited understanding of its pathophysiology and lack of effective treatments [18, 19].

Chinese herbal medicine has been widely used in the treatment of people with cancer in China [20]. Studies have shown that Chinese herbal medicine has beneficial effects on assisting in treating cancer, retarding cancer progression, boosting the immune system, and ameliorating chemotherapy-induced complications and side effects, such as pain and fatigue [21, 22].

Shenmai injection (SMI), derived from the famous traditional Chinese herbal prescription Shendongyin which is described in the *Zheng Yin Mai Zhi* [23] by Qin Changyu in the Ming dynasty, is extracted from red ginseng (*Radix ginseng Rubra*) and dwarf lilyturf tuber (*Radix ophiopogonis*). It has been widely used in traditional medicine in China and has been developed as a traditional Chinese patent drug based on the national standards approved by the CFDA (China Food and Drug Administration) since 1995.

In traditional Chinese medicine (TCM), *qi* usually refers to life energy, which manifests simultaneously on the physical and mental-spiritual levels [24]. The quintessence of TCM is syndrome differentiation and treatment. From the perspective of TCM, CRF is recognized as deficiency syndrome pattern, which is mainly caused by deficiency of *qi* and disharmony of *yin* and *yang* [25]. Many patients with CRF have symptoms of fatigue, listlessness, shortness of breath, and weak pulse after chemotherapy. These symptoms are similar to the syndrome of *qi* deficiency in TCM theory. According to TCM theory, this herbal prescription has been identified as an effective medication to tonify *qi* for reducing general weakness [23].

Ginsenosides, extracted from ginseng, and ophiopogonis, extracted from dwarf lilyturf tuber, are the principal constituents responsible for the pharmacological activities in SMI [26]. There are several studies on ginseng for the treatment of fatigue, and studies in mice have shown that ginseng can alleviate fatigue [27, 28]. One large study evaluating ginseng in combination with vitamins and minerals in 232 patients who had functional fatigue for over 10 years concluded that the ginseng formula improved fatigue symptom scores statistically significantly more than vitamin placebo [29]. An abstract presented at the 2003 meeting of the American Society of Clinical Oncology evaluated ginseng in people with cancer. It reported that ginseng significantly improved total and average fatigue levels, even though the sample size was quite small (*n* = 20) [30].

This study aims to assess the efficacy and safety of the addition of SMI to conventional therapy for advanced NSCLC patients undergoing chemotherapy. The primary hypothesis is that the SMI plus conventional therapy (here our treatment group) will experience less fatigue, as measured using Functional Assessment for Chronic Illness Therapy-Fatigue (FACIT-F) subscale scores, than conventional therapy alone (here our control group). Secondary objectives are to evaluate the efficacy of SMI on the following factors: symptom distress scores, depression, sleep disorders, quality of life (QOL), levels of inflammatory indicators, and adverse events (AEs).

## Methods/design

### Design

**Fig. 1:**
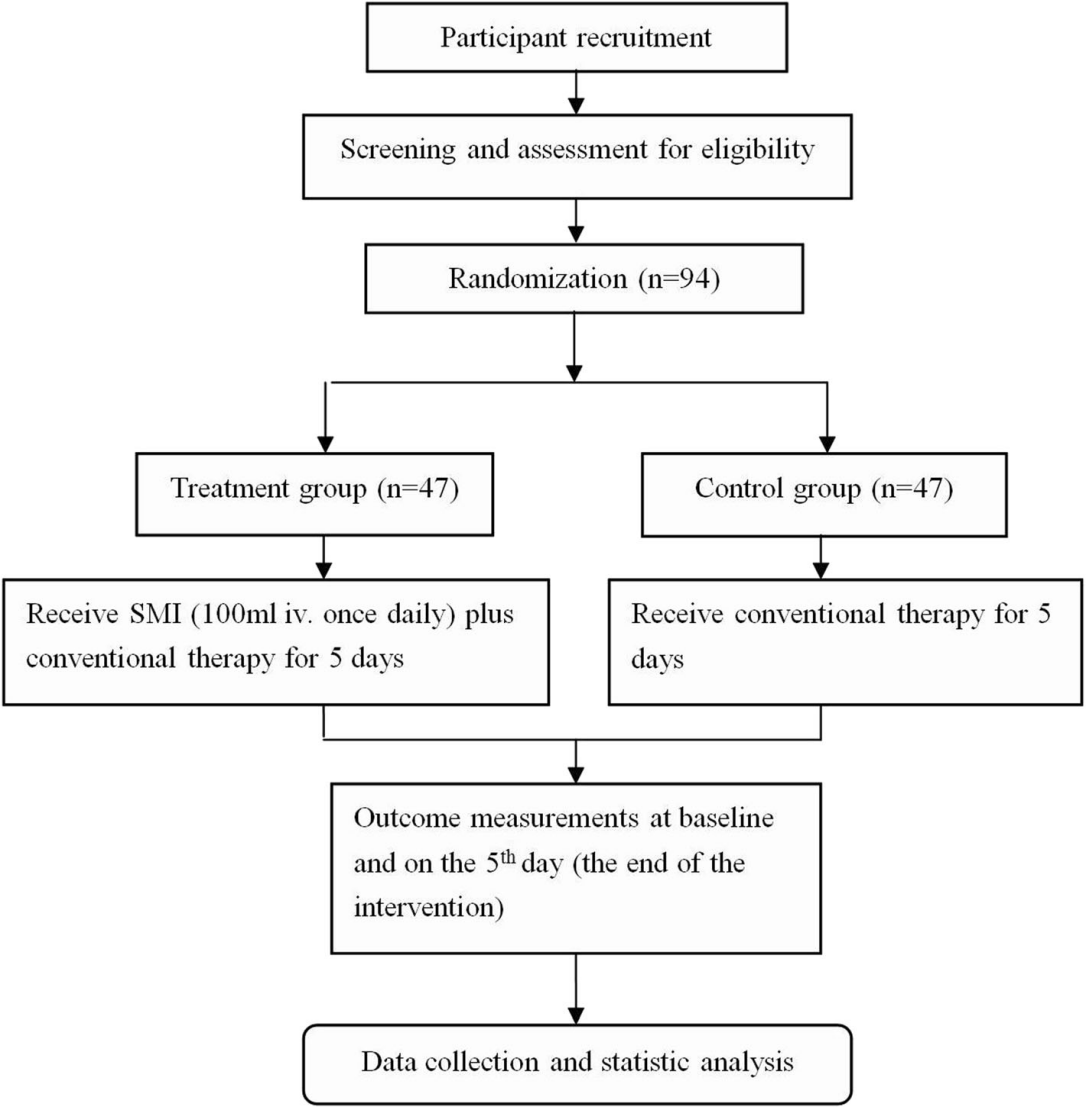
Study design flow chart. iv. intravenous, SMI Shenmai injection

**Fig. 2:**
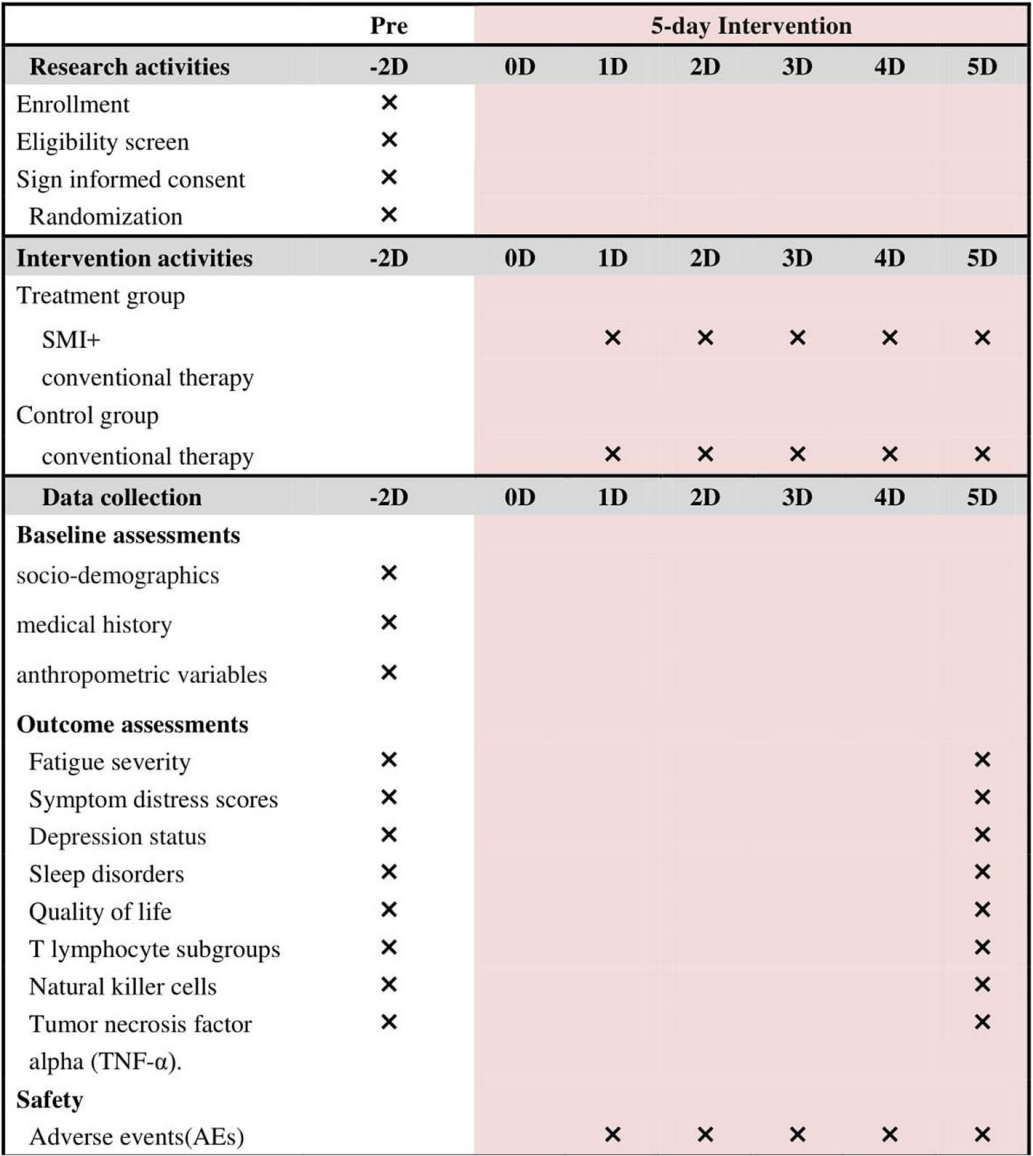
Treatment schedule and outcome measures. D day, SMI Shenmai injection

This study is registered on chictr.org.cn (ChiCTR-INR-17013737). A brief flowchart of the entire study is shown in Fig. 1, and the schedule of events is provided in Fig. 2. This will be a two-group, prospective, randomized controlled trial (RCT) to evaluate the efficacy and safety of SMI for CRF NSCLC patients undergoing chemotherapy. Eligible participants will be randomized to either a treatment group receiving a 5-day SMI regimen plus conventional therapy or a control group receiving only the conventional therapy. The protocol includes the recommended elements elaborated upon in the Standard Protocol Items: Recommendations for Interventional Trials (SPIRIT) [31] checklist (Additional file 1).

### Setting and participants

The study will be conducted in Guangzhou, the third largest city in southeastern China. Patients will be recruited from the oncology inpatient department at each of the three GPHCM branch hospitals in the different urban districts of Guangzhou. A complete list of inclusion and exclusion criteria is provided in the following section.

### Randomization, allocation concealment, and blinding

Patients who provide informed consent will be randomized into either a treatment group receiving a 5-day SMI regimen plus conventional therapy or a control group receiving only the conventional therapy. A block randomization sequence will be generated by SAS 9.2 (SAS Institute Inc., Cary, NC, USA) in a 1:1 ratio. Treatment assignments will be conducted using a web-based allocation system. Given the nature of the intervention, it is impossible to blind the patients and personnel involved in conducting the programs. Outcome assessors, laboratory technicians, data managers, and statisticians will be unaware of the treatment allocations.

### TCM syndrome pattern differentiation

The diagnosis as described in TCM, deficiency of *qi* syndrome pattern, will be based on guidelines delineated in the Clinical Research of New Investigational Drugs in Traditional Chinese Medicine [32]. The diagnostic criteria are as follows: 1) primary signs and symptoms include fatigue, listlessness, shortness of breath and weak pulse; and 2) secondary signs and symptoms include soreness of the lower back and knees, spontaneous perspiration, and pale lips.

Participants will be diagnosed with deficiency of *qi* syndrome if they have two or more of the primary signs or symptoms, at least one of the secondary signs or symptoms, and examination of the tongue and pulse indicates deficiency of *qi*. After being screened for inclusion and exclusion criteria, eligible patients will be entered into the trial.

### Eligibility criteria

#### Inclusion criteria

Participants who meet the following criteria are eligible:

1. Patients with histologically or cytologically confirmed NSCLC who are undergoing chemotherapy;
2. Clinical stage IIIB, IIIC, or IV;
3. Clinically confirmed CRF at a score of ≥ 4 on the Edmonton Symptom Assessment Scale (ESAS; scale of 0 to 10) within the past 24 h;
4. Chinese syndrome differentiation of *qi* deficiency;
5. Aged between 18 and 75 years;
6. Eastern Cooperative Oncology Group (ECOG) performance status score 1 and 2;
7. Able to read Chinese and willingness to provide signed informed consent;
8. Hemoglobin ≥ 90 g/L.

#### Exclusion criteria

Exclusion criteria are as follows:

1. Active double cancer;
2. Severe mental disorder (severe anxiety, depression, or cognitive dysfunction);
3. Severe central nervous system metastasis or acute disease interfering with attendance for therapy;
4. Impaired hepatic (alanine aminotransferase level ≥ 80 IU/L) or renal (creatinine level ≥ 2 mg/dL) function;
5. Received erythropoietin (EPO) and proprietary Chinese medicine reinforcing *qi* orally or intravenously;
6. A history of hypersensitivity to the components of the trial medication;
7. Pregnant or lactating women;
8. Unable to understand or sign an informed consent form.

### Interventions and control

The treatment delivery processes for both the intervention and control groups are outlined in Fig. 2.

#### Control group

Patients in the control group will receive only conventional therapy according to national guidelines.

#### Intervention group

Patients in the treatment group will receive a 5-day Shenmai injection regimen plus conventional therapy. According to national guidelines, conventional therapy includes the routine use of antiemetic and gastro-protective agents. SMI will be manufactured by Huarun Ya’an Sanjiu Pharmaceutical Co., Ltd., based on good manufacturing practice (GMP) standards. Each milliliter of the injection is equivalent to 0.1 g of red ginseng (*Radix ginseng Rubra*) and 0.1 g dwarf lilyturf tuber (*Radix ophiopogonis*) of crude drug. It will be administered at a dose of 100 ml by intravenous infusion once daily for 5 days as a period. Treatment will be administered by Chinese medicine physicians with 5 years of formal university training in Chinese medicine, a license to administer treatment, and at least 1 year of clinical experience.

### Data collection

The study data collection process is outlined in Fig. 2.

### Baseline data

The following baseline data will be collected using questionnaires and a review of medical charts: 1) sociodemographics; 2) medical history; and 3) anthropometric variables.

### Outcome measures and assessments

Each participant will be asked to attend an in-person assessment appointment at the GPHCM Oncology Department at two time points: baseline and day 5 (the end of the intervention). During each assessment, participants will be asked to complete specified physiological tests and self-reported questionnaires. All tests will be administered by one of two trained independent assessors.

### Primary outcome

#### Fatigue severity

Fatigue severity will be self-assessed using the Functional Assessment for Chronic Illness Therapy-Fatigue (FACIT-F) subscale at baseline and on day 5. FACIT-F is a well-validated quality-of-life instrument widely used for the assessment of CRF in clinical trials. It consists of 27 general quality-of-life questions divided into four domains (physical, social, emotional, and functional) and the 13-item FACIT-F fatigue subscale [33]. FACIT-F allows patients to rate the intensity of their fatigue and its related symptoms on a scale of 0 to 4 (0, not at all; 4, very much). The total score for FACIT-F is the sum of the four domains (physical, social, emotional, and functional) plus the fatigue subscale.

### Secondary outcomes

CRF most commonly occurs with other symptoms, such as depression, anxiety, pain, sleep disorder, and loss of functional status [7]. CRF profoundly impacts on a person’s physical, emotional, and mental well-being [11]. The secondary outcomes are listed below.

#### Symptom distress scores

Symptom distress scores will be self-assessed using the ESAS [34] at baseline and on day 5. This validation instrument will assess nine symptoms commonly experienced by cancer patients during the previous 24 h: pain, fatigue, nausea, depression, anxiety, drowsiness, dyspnea, anorexia, and well-being [35, 36]. These tools have been validated and are reliable for assessing the intensity of symptoms in patients with cancer [33, 34, 37].

#### Depression status

Depression and anxiety will be self-administered by the Hospital Anxiety and Depression Scale (HADS) at baseline and on day 5. This scale has been shown to be a valid and reliable measure of the severity of emotional disorders and is used in general hospital practice. Participants will be instructed to choose one response from a list of pre-provided answers that best describe their current feelings. The HADS is a 14-item self-report screening scale originally developed to indicate the presence of anxiety and depressive states in the setting of a medical outpatient clinic and is widely used to assess patients with advanced disease [38].

#### Sleep disorders

Sleep disorders will be self-administered using the Pittsburg Sleep Quality Index (PSQI) at baseline and on day 5. This questionnaire has been used as an effective instrument for measuring the quality and patterns of sleep. Several studies using the PSQI have confirmed its high level of validity and reliability [39].

#### Quality of life

Quality of life will be self-administered using the Functional Assessment of Cancer Therapy-lung (FACT-L) questionnaire. The FACT-L questionnaire measures 44 items rated on five subscales. This questionnaire has been used previously to measure QOL in the last 7 days for lung cancer patients, with each subscale using a 0 to 4 Likert scale. The four subscales (emotional, functional, physical, and social) query patient’s well-being. The fifth subscale queries the patient’s pulmonary (lung) functionality [40].

#### Levels of immunologic indicators

Blood samples will be collected at baseline and day 5 (the end of intervention) to measure the levels of immunologic indicators. T lymphocyte subgroups, natural killer cells, and TNF-α will be analyzed in the GPHCM laboratory. These are associated with postchemotherapy fatigue in patients [17].

### Safety

An independent Data and Safety Monitoring Committee will evaluate the progress of the study and assess the safety data, as requested during the study. Adverse events (AEs) will be defined as any undesirable experience participants endure during the trial period, regardless of whether or not it is associated with the intervention. Participants will be instructed to report AEs to the research team, and a research nurse will monitor participants for potential occurrences of AEs. All details of AEs, such as time of occurrence, severity, management, and causality to the intervention, will be recorded on case report forms. All AEs will be followed up from the date they are brought to the investigator’s attention until resolution. Severe AEs must be reported to both the GPHCM Data and Safety Monitoring Committee and the GPHCM Ethics Committee within 24 h. Severe AEs will be defined according to the International Conference on Harmonization guidelines [41]; any adverse event will be regarded as serious if it results in death, is life-threatening, requires hospitalization or prolongation of existing hospitalization, or results in persistent or significant disability or incapacity.

To assess safety, we will perform the following tests on participants at the screening phase (baseline) and on day 5 (the end of the intervention): routine blood, routine feces, routine urine, liver, and kidney function, and electrocardiography. Safety will be assessed according to the National Cancer Institute Common Terminology Criteria for Adverse Events, version 4.0.

In particular, we will pay attention to adverse drug reactions (ADRs) associated with SMI. Because of its effectiveness, the scope of diseases treated with SMI has gradually broadened, and reports of associated adverse reactions have increased correspondingly. The number of published literatures has ranked SMI ninth for ADRs in 33 varieties of Traditional Chinese Medicine injections in the 2004 edition of the National Essential Drugs List (2004 edition) in China [42].

Current evidence shows that SMI has a lower rate of ADRs. However, a systematic review of ADRs associated with SMI since 1995 showed that ADR cases were mainly systemic reactions (allergic shock), skin lesions (urticaria and pruritus), and local pain [43]. Because most ADRs occurred in the first 30 min of the first SMI administration, we suggest that patients should be closely observed during this period. Meanwhile, for patients who had continuous treatment with SMI, there is a need for close attention. ADR severity for each event as classified according to World Health Organization guidelines is shown in Table 1 [44].

**Table 1:** Classification of adverse drug reaction (ADR) severity for Shenmai injection

Classification of ADR severity	
Class I	ADRs are deadly or life-threatening; patients need to be withdrawn from drugs and treated immediately; or ADRs last for more than 1 month.
Class II	Patients have pathologic and physiologic changes and must be withdrawn from drugs and treated; or ADRs last for more than 7 days.
Class III	Patients cannot endure the ADRs and must be given a lower dose or withdrawn from drugs; patients improve after symptomatic treatment.
Class IV	Patients can endure the ADRs, and no lowering of dose or drug withdrawal is necessary. Whether without treatment or through symptomatic treatment, patients improve

### Sample size estimation

The primary endpoint is the fatigue severity self-assessed using the FACIT-F subscale. The sample size is calculated based on the primary endpoint. Previous clinical practice suggests a mean difference (MD) of 4.95 between groups, and the same standard deviation (SD) of 7.28 for each group. Using a superiority test, it is estimated that 38 participants per group are needed to achieve 90% power and a (two-sided) 5% significance level in detecting treatment differences. Thus, the final sample size has been set at a total of 94 patients (47 in each group), assuming a 20% dropout rate.

### Statistical analysis

Efficacy and safety analyses will be conducted according to the intention-to-treat principle. All statistical analyses will be performed using Statistical Packages of Social Sciences software (SPSS; version 19.0). All results made in this study are based on two-sided tests. A *P* value < 0.05 will be considered statistically significant. Continuous data will be presented as means and standard deviations and compared using the independent *t* test or Wilcoxon’s rank sum test, while categorical data will be presented as percentages or frequencies and compared using the chi-square or Fisher’s exact test. Data for subjects who meet the dropout criteria (i.e., incidence of serious AEs, < 80% compliance with the protocol, incomplete data that could influence the trial, reluctance to continue the study, large error in protocol, or deviation from the protocol) will be excluded. Missing values will be implemented by multiple imputations.

### Quality control and trial management

The management structure comprises principle investigators (PI), a Trial Management Group, a Data Monitoring Committee, and a Trial Steering Committee. The Trial Management Group is responsible for the day-to-day delivery and for conducting the trial. It will comprise a project manager and investigators from the GPHCM. The Trial Management Group will meet weekly to discuss trial progress. The project manager will have a face-to-face meeting with the PI every other week. The role of the Data Monitoring Committee is to review safety and efficacy data and to make recommendations to the Trial Steering Committee. It will comprise four fully independent members: one chairman, one oncologist, one senior biostatistician, and one clinical pharmacology expert from the GPHCM. The Data Monitoring Committee will meet once, prior to the start of patient recruitment, and at least once a year for those years in which patients are involved in exercise sessions. The Trial Steering Committee is responsible for approval of any amendments to the main study protocol and, to monitor the trial, steering it towards its overall objectives, to approve and comment on project deliverables, to consider the recommendations of the Data Monitoring Committee, and to resolve any problems brought up by the Trial Management Group. It will comprise one independent chairman, Dr. Wanyin Wu, Dr. Xiaobing Yang, Dr. Hong Deng, and two other independent members including at least one patient and a public involvement representative. Responsibility for calling and organizing the Trial Steering Committee meetings lies with the PI. The Trial Steering Committee will meet at least annually, more frequently if needed. Representatives of the Data Monitoring Committee are to be invited to all Trial Steering Committee meetings. Before recruitment, the whole research team, including investigators, research assistants, and research nurses will be required to attend a training workshop. This will be done before the trial to ensure their strict adherence to the study protocol and familiarity with the trial administration process. The data collected in this trial will comprise information recorded in case report forms and questionnaires.

Data will be entered using the double-entry method. Data quality will be checked regularly by research assistants and overseen by monitors. Data monitoring will be conducted regularly with standard operation procedures by a team from the GPHCM Key Unit of Methodology in Clinical Research. Inspections will be performed regularly by the GPHCM Department of Science Research. All modifications are to be marked on the case report forms. Data managers will then recheck the data before logging it. The database will be locked after all data have been cleaned. If participants withdraw from the trial during the study period the reasons must be documented, and the dropout rate is to be statistically analyzed.

## Discussion

CFR is a common and unavoidable side effect of current forms of cancer treatment. It interferes with daily activities of cancer patients and affects various aspects of life, including physiological, social, and psychological well-being [45]. The National Comprehensive Cancer Network provides an evidence-based guideline that includes pharmacological and nonpharmacological interventions [46]. However, it does not include TCM, which relieves the symptoms of CRF effectively. The use of Chinese herbal injections for treatment of CRF should adopt an evidence-based approach.

This study has some limitations. First, due to the nature of the intervention, blinding is impossible. We will make every effort to ensure that outcome assessors, laboratory technicians, data managers, and statisticians are unaware of the treatment allocations. Another limitation is the 5-day treatment period which does not have a follow-up. While many studies use 1- or 2-week treatment periods in the clinical trial setting [47, 48], it would be appropriate in future studies to administer treatment for longer periods with long-term follow-up research since fatigue is a chronic symptom and, in most cases, affects patients for far longer than several weeks. This could make the results more convincing.

Considering that SMI originated and developed mainly from the traditional medicinal system in China, which considered the constitution or specific characteristics of health status in prescribing medication, the concurrent use of outcome measurements reflecting the perspective of traditional medicine will be of interest in future studies. In traditional medicine in China, SMI is not prescribed for everyone but for those with *qi*-deficiency syndrome, which is defined as a hypersensitive, easily fatigable state [49]. Although fatigue is one of the typical symptoms of this *qi*-deficiency syndrome, other factors that constitute the syndrome may also be considered in studying SMI.

Despite its limitations, we believe that this study has the potential to contribute to the development of an effective intervention to help relieve CRF. It will also establish feasibility and provide preliminary evidence on the efficacy and safety of SMI in advanced NSCLC patients with CRF. Based on the results of the study, future studies should employ more subjects, establish an appropriate control group, and include long-term administration and follow-up.

### Trial status

Recruitment started in December 2017, and it is expected to finish in April 2019.

## Additional file

Additional file 1: SPIRIT checklist. (DOC 105 kb)

## Abbreviations

ADR: Adverse drug reaction
AE: Adverse event
CRF: Cancer-related fatigue
ECOG: Eastern Cooperative Oncology Group
ESAS: Edmonton Symptom Assessment Score
FACIT-F: Functional Assessment for Chronic Illness Therapy-Fatigue
FACT-L: Functional Assessment of Cancer Therapy-lung
GMP: Good manufacturing practice
HADS: Hospital Anxiety and Depression Scale
NSCLC: Non-small cell lung cancer
PSQI: Pittsburg Sleep Quality Index
QOL: Quality of life
RCT: Randomized controlled trial
SMI: Shenmai injection
TCM: Traditional Chinese medicine
TNF: Tumor necrosis factor
